# Whole-Genome Sequencing Analysis to Identify Infection with Multiple Species of Nontuberculous Mycobacteria

**DOI:** 10.3390/pathogens10070879

**Published:** 2021-07-11

**Authors:** Visal Khieu, Pimjai Ananta, Orawee Kaewprasert, Marut Laohaviroj, Wises Namwat, Kiatichai Faksri

**Affiliations:** 1 Department of Microbiology, Faculty of Medicine, Khon Kaen University, Khon Kaen 40002, Thailand; visal.cn@gmail.com (V.K.); pimjais@kku.ac.th (P.A.); k_orawee@kkumail.com (O.K.); marut@kku.ac.th (M.L.); wisnam@kku.ac.th (W.N.); 2 Research and Diagnostic Center for Emerging Infectious Diseases (RCEID), Faculty of Medicine, Khon Kaen University, Khon Kaen 40002, Thailand; 3 Clinical Laboratory Unit, Srinagarind Hospital, Faculty of Medicine, Khon Kaen University, Khon Kaen 40002, Thailand

**Keywords:** mixed species infection, nontuberculous mycobacteria, NTM, whole-genome sequencing

## Abstract

Mixed infection with multiple species of nontuberculous mycobacteria (NTM) is difficult to identify and to treat. Current conventional molecular-based methods for identifying mixed infections are limited due to low specificity. Here, we evaluated the utility of whole-genome sequencing (WGS) analysis to detect and identify mixed NTM infections. Analytical tools used included PubMLST, MetaPhlAn3, Kraken2, Mykrobe-Predictor and analysis of heterozygous SNP frequencies. The ability of each to identify mixed infections of NTM species was compared. Sensitivity was tested using 101 samples (sequence sets) including 100 *in-silico* simulated mixed samples with various proportions of known NTM species and one sample of known mixed NTM species from a public database. Single-species NTM control samples (155 WGS samples from public databases and 15 samples from simulated reads) were tested for specificity. Kraken2 exhibited 100% sensitivity and 98.23% specificity for detection and identification of mixed NTM species with accurate estimation of relative abundance of each species in the mixture. PubMLST (99% and 96.47%) and MetaPhlAn3 (95.04% and 83.52%) had slightly lower sensitivity and specificity. Mykrobe-Predictor had the lowest sensitivity (57.42%). Analysis of read frequencies supporting single nucleotide polymorphisms (SNPs) could not detect mixed NTM samples. Clinical NTM samples (*n* = 16), suspected on the basis of a 16S–23S rRNA gene sequence-based line-probe assay (LPA) to contain more than one NTM species, were investigated using WGS-analysis tools. This identified only a small proportion (37.5%, 6/16 samples) of the samples as mixed infections and exhibited only partial agreement with LPA results. LPAs seem to be inadequate for detecting mixed NTM species infection. This study demonstrated that WGS-analysis tools can be used for diagnosis of mixed infections with different species of NTM.

## 1. Introduction

Nontuberculous mycobacteria (NTM) are environmental mycobacteria that have been associated with human diseases since the 1930s [1]. NTMs are mostly opportunistic pathogens, becoming true pathogens in immunocompromised patients such as HIV patients [2]. NTMs are difficult to treat and diagnostically easily confused with the more common disease, tuberculosis. Moreover, mixed infections with different strains or species of NTM can occur [3,4]. Disease status [5] and treatment outcomes in people infected with multiple NTMs can differ from those with individual infections [4,6]. Misdiagnosis and inappropriate treatment is a common critical problem in multiple-NTM infections [7]. Cases of multiple-species NTM infections have been reported in many countries [3,4,8,9]. Such infections may be pulmonary [4], cutaneous [8] or disseminated [10]. Accurate identification of mixed species infection will facilitate appropriate antibiotic treatment.

Conventional methods for NTM species identification include biochemical tests and use of phenotypic characteristics. These methods are time-consuming and sometimes cannot provide accurate identification [11]. Molecular methods based on the line-probe assay (LPA) and real-time PCR can be used for more accurate NTM species identification. In addition, also useful is sequencing of specific target genes including 16S rRNA [12], *rpoB* [13] and the *hsp65* gene [14]. The inability of a single gene to distinguish between genetically similar mycobacterial species has resulted in the use of multigene sequencing methods [15]. In addition, there is no gold standard for detection and identification of multiple species infection of NTM. LPA and multiplex real-time PCR/melting curve analysis have been applied to detect mixed infections with different NTM species based on the 16S-23S rRNA intergenic transcribed spacer (ITS) region and achieved good performance using material directly from clinical samples [16,17,18]. However, the resolution of these techniques is still low because not all NTM species, especially newly discovered species, have not been included in assay development. Therefore, accurate diagnosis of mixed infection requires use of another high-resolution method for confirmation.

The advent of whole-genome sequencing (WGS) allows the sequencing of the entire genome of microorganisms and has ability to sequence multiple species in samples from one individual [19]. Free WGS-analysis tools for species identification and diagnosis of many pathogens [20], including mycobacteria, are increasingly available. WGS analysis has been successfully used to identify infection caused by multiple strains of *M. tuberculosis* [21,22]. Mixed infection with different species of NTMs have generally been detected only incidentally [23,24]. No study has systematically evaluated and applied bioinformatics analysis approaches for the identification of infections due to multiple species of NTMs.

Here, we aim to evaluate the performance of WGS-analysis approaches for identification of mixed infections of NTMs. We use *in-silico* simulated mixtures with various proportions of known NTM species, WGS analysis of single and mixed NTM species datasets, and datasets from clinical samples of mixed NTM species (as detected by LPA) to evaluate the performance of several WGS-analysis approaches.

## 2. Results

### 2.1. Detection and Identification of Mixed Infections from In-Silico Simulated Mixed-Species NTM Dataset

**Figure 1:**
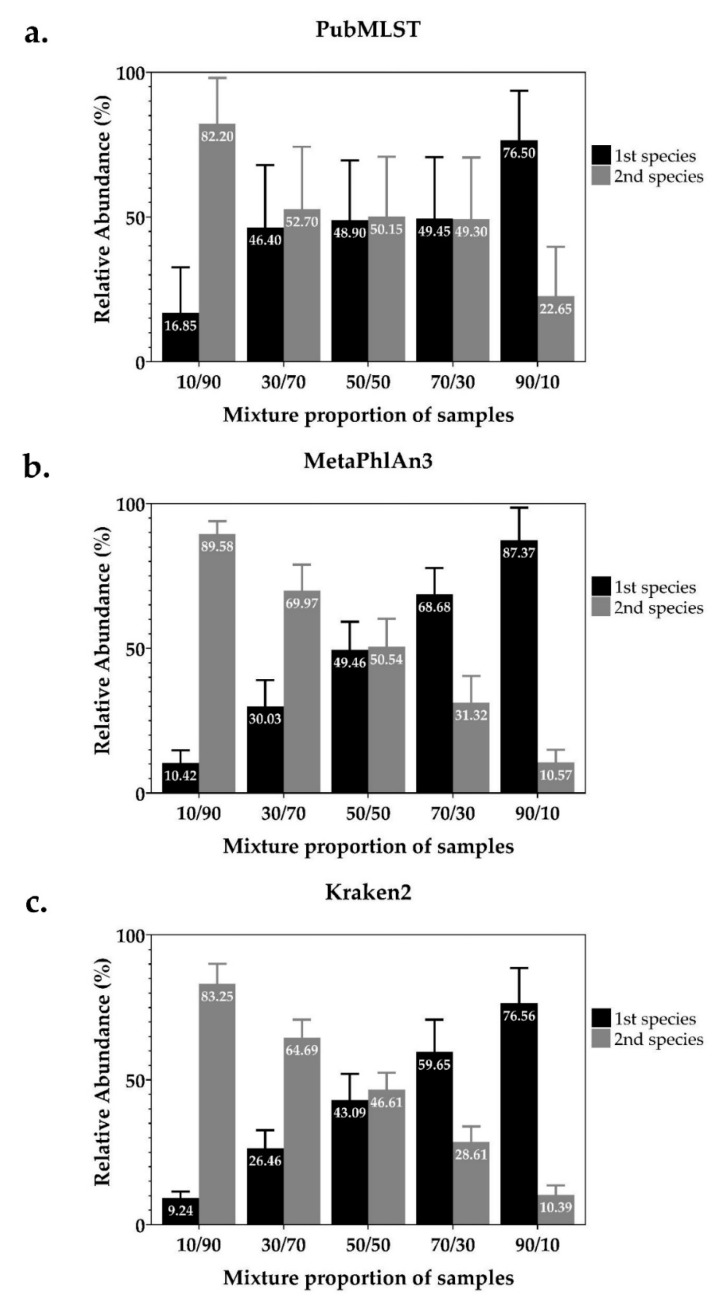
Average relative abundance (%) of each NTM species presented in simulated mixed samples were estimated by (**a**) PubMLST, (**b**) MetaPhlAn3 and (**c**) Kraken2. (**a**) One sample was identified as a single species. (**b**) One simulated mixed NTM species sample levels and five samples were excluded.

We used the simulated mixed NTM species dataset (20 mixed samples, each with five different ratios of reads of the two species, *n* = 100) (Table S1), to test the ability of four WGS-analysis tools to recognize mixed infections and identify the species present (Table 1). Kraken2 achieved a perfect score. MetaPhlAn3 can detect species present in all 100 samples, but 5 (5%) samples were incorrectly reported as a mixture between *M. tuberculosis* and *M. canettii* that belong to the same species complex. PubMLST correctly identified species represented in 99 samples but failed to identify one sample which consisted of a 10/90 ratio of reads from *M. fortuitum* and *M. peregrinum*. All samples containing the *M. abscessus* complex were correctly identified into subspecies using MAB-MLST (additional tool of PubMLST). Mykrobe-Predictor correctly identified species in only 58 samples (58%), incorrectly claimed that a single species was present in 17 samples (17%) and incorrectly identified the species present in 25 mixed samples (25%), but mostly suggested species within the same species complex. Apart from Mykrobe-Predictor, all tools reported the expected proportion of each species: MetaPhlAn3 and Kraken2 were the most accurate (Figure 1).

The performance of the WGS-analysis tools for detection and identification of NTMs was also assessed using WGS data for 15 single-species datasets (processed through the ART simulator) from GenBank (Table S2). Both Kraken2 and PubMLST provided 100% specificity (*n* = 15). Subspecies identification of the *M. abscessus* complex were correctly reported by MAB-MLST in all samples. MetaPhlAn3 and Mykrobe-Predictor correctly identified the single species represented in 14 (93.33%) datasets. The former method claimed that one dataset contained a mixture of *M. canettii* and *M. tuberculosis* and the latter misidentified the species present as *M. intracellulare*.

Besides the four WGS-analysis tools, we tried using the analysis of read frequencies supporting SNPs to identify species in simulated mixed-sequence samples of *M. intracellulare* and *M. abscessus*. Various proportions of the two species were represented in these simulated samples. However, there was no clear pattern of allele frequencies at SNP sites that made it possible to identify the number and proportions of each species represented (Figure S1). This approach was therefore not included in later comparisons.

### 2.2. Detection and Identification of NTM Species in Mixed Infections: Dataset from GenBank

We retrieved publicly available WGS data for one known mixed-species NTM sample (*M. hassiacum* and *M. peregrinum*), obtained from a bird. All WGS-analysis tools correctly reported both species to be present, except that Mykrobe-Predictor identified only one species (*M. hassiacum*) (Table 2). From metagenomic assembly analysis, of the three specific target genes, only the *rpoB* gene was found. This was represented by two sequence types, one with 100% similarity to *M. hassiacum* and one with 98.34% similarity to *M. peregrinum* (Table 2 and Table S3).

WGS datasets (*n* = 155), each of a known single mycobacterial species, from a public database were used as single-species controls (Table S4). Kraken2 provided the highest specificity by correctly identifying the species in 152/155 (98.06%) datasets, followed by PubMLST (149/155, 96.12%), Mykrobe-Predictor (149/155, 96.12%) and MetaPhlAn3 (128/155, 82.58%) (Table 3). All four analysis tools correctly identified the same 120/155 (77.41%) samples. Notably, a sample of *M. intermedium* was correctly identified by Kraken2, but the other tools failed to do so. MAB-MLST correctly differentiated subspecies within the *M. abscessus* complex in 10/16 (62.5%) cases as *M. abscessus* and in 9/25 (36%) cases as *M. abscessus* subsp. *massiliense*.

### 2.3. Comparison of the Four WGS-Analysis Tools for Identifying Mixed Infections with Different NTM Species

The sensitivity and specificity of the four WGS-analysis tools for detection and identification of species present in the simulated mixed NTM datasets and samples from a public database are shown (Table 4). Kraken2 had the highest overall sensitivity and specificity (100%, 98.23%). PubMLST exhibited the second-highest sensitivity and specificity (99%, 96.47%), followed by MetaPhlAn3. The sensitivity of Mykrobe-Predictor was very much lower (Table 4).

### 2.4. Concordance between WGS-Analysis Tools and LPA for Detection and Identification of Mixed NTM Species in Clinical Samples

Sixteen clinical samples of mixed infection with different species of NTM (identified using LPA as the standard) were used to evaluate the performance of WGS-analysis tools (Table 5). MetaPhlAn3 was the most successful at detecting mixed infections (6/16, 37.5%). In three of these cases, MetaPh1An3 correctly identified one of the species present and in the remaining cases none of the species reported was in agreement with the result from LPA. A further feature of the MetaPhlAn3 result was that, in three datasets, at least one species was represented by fewer than 0.2% of the reads (Table 6). The remaining tools inferred the presence of mixed infections in only a single sample each (6.25%), but without agreement among them. Even when disagreeing with the results of the LPA, the WGS-analysis tools often agreed with each other as to the identity of at least one species in a sample (Table 6).

The metagenomic assembly analysis could detect only two genes; 16S rRNA and *rpoB*. In the positive control (known mixed NTM dataset), the two sequence types of *rpoB* were found, indicating mixed NTM species. Only one clinical sample (1/16, 6.25%) of suspected mixed-NTM according to LPA yielded the concordant mixed sequence types (*M. tuberculosis* with 100% similarity and *Mycobacterium* sp. *MOTT-01* or M. *parascrofulaceum* with 94.20% similarity) (Tables S3 and S5). 

## 3. Discussion

WGS analysis is increasingly being used for clinical laboratory diagnosis of bacterial infections [25,26] including those due to NTMs [23]. However, no study has previously evaluated WGS-analysis approaches for detection and classification of mixed-species NTM infections. Here, we have demonstrated that particular WGS-analysis tools can be used for this purpose.

In clinical samples, it is difficult to detect mixed infections of NTM species and to identify the species involved. The current approach, using molecular probes specific for a single gene target, has limitations, especially when it comes to species identification [16,17]. The presence of more than one species of NTM in cultured specimens might not be apparent from colonial morphologies. In this study, we analyzed WGS data from cultures identified as including more than one species of NTM, as diagnosed by routine laboratory analysis using LPA.

We assessed preexisting WGS-analysis tools including the metagenomics approaches (MetaPhlAn3 and Kraken2), web-based species identification relying on ribosomal multilocus sequence typing (PubMLST) and a drug-resistance prediction tool for *M. tuberculosis* (Mykrobe-Predictor). An analysis pipeline based on the read frequencies supporting SNPs, which has been used to detect mixed strain infection [22], was also included.

There is as yet no gold standard for detection and classification of mixed NTM infections. To ensure that we had accurate identification of the species involved, we produced simulated WGS datasets (*n* = 100) that included reads from various pairs of mycobacterial species (*n* = 15) frequently found in the clinical setting and various ratios of reads from the two species. WGS data (*n* = 155) of single species of common pathogenic NTMs, from a public database, were included for comparison. To ensure the purity of single-species control data, the simulated sequence reads were generated from reference genome data for each species (*n* = 15). A WGS dataset of known mixed NTMs isolated from a bird was also included. Finally, the WGS-analysis tools were assessed using data from clinical samples (*n* = 16) suspected (on the basis of LPA analysis) to contain more than one NTM species.

First, we tried the approach of analyzing SNP allele frequencies to identify the mixed NTM species represented in the simulated dataset. This approach has been successfully used to identify mixed-strain infections of *M. tuberculosis* [22] using WGS data. We could not find appropriate clustering patterns of alleles (Figure S1). We tried to map the sequence reads to reference genomes of different species, but could not identify mixed NTM species. This could be due to the higher diversity among full species compared with subspecies analysis, or to the greater complexity of the genome sequences of NTM compared to *M. tuberculosis* [27].

Expecting that they might perform better, we then evaluated well-known metagenomics analysis tools including Kraken2 and MetaPhlAn3. Kraken2 identifies pathogens based on exact *k-mers* alignment [28] and has been successfully applied to detect food-borne and vector-borne pathogens in clinical samples [29,30]. A previous study used Kraken2 to classify NTM isolates into correct species and the results were found to be concordant with conventional PCR and direct sequencing [31]. Here, we found Kraken2 to have high sensitivity (100%) and specificity for detecting and identifying NTMs in mixed infections. Furthermore, Kraken2 correctly identified the proportions of the minor species in the mixture. The specificity of Kraken2 based on single-species controls was 98.23%: misidentification of three species might have been due to limitations of the database associated with the tool or to the presence of closely related species. Both the database and the algorithm of each analysis tool plays a role in its performance for species identification [30,32,33]. The higher performance of Kraken2 might be due to its use of an exact *k-mer* alignment to the *k-mer* of the lowest-common ancestor (LCA) of the taxa and higher specificity due to the default option of *k* = 35. However, differentiating subspecies of *M. abscessus* that are associated with different drug-resistance patterns [34,35] remains a challenge for Kraken2. One sample of *M. intermedium* was correctly identified by Kraken2, whereas other tools failed to do this. Overall, Kraken2 provided the most reliable results for identifying single-species controls.

MetaPhlAn3 is another metagenomics tool that can identify and estimate the proportions of members of a microbial community using unique clade-specific marker genes [36]. Previously, this tool has been applied to identify the composition of bacterial species in the intestine and skin [37,38]. However, had no previous study has assessed the performance of this tool for identifying mixed-species NTM infections. Here, we found that MetaPhlAn3 had high sensitivity (95.04%). Its main failing was in confusing *M. tuberculosis* with *M. canettii*, species that belong to the same complex. This tool could also reliably estimate the relative abundance of each species represented in the dataset. The specificity of MetaPhlAn3 was 83.52%, due in part to its failure to distinguish between closely related species in the same species complex [39]. Similarly, subspecies of *M. abscessus* could not be identified by this tool.

Previously, PubMLST has been used for NTM detection and identified species correctly (*n* = 29) when compared to other methods including MLSTverse [40]. However, this previous study investigated only samples with single NTM species. Here, we demonstrated that PubMLST can be used to identify species in mixed NTM infections with high sensitivity (99%) and specificity (96.47%). In one simulated mixed sample consisting of reads from *M. fortuitum* (10%) and *M. peregrinum* (90%), only the latter species was identified. Six samples of single-species controls were misidentified as incorrect species or as mixed species. In addition, PubMLST could not identify *M. abscessus* at the subspecies level. An additional MLST-based tool is available for this purpose (MAB-MLST [41]). This extended web-based tool provided only moderate performance for identifying the members of the *M. abscessus* complex due to limitations of the specific gene profiles or database.

Mykrobe-Predictor [42] was developed to predict drug-resistance in *M. tuberculosis* and *Staphylococcus aureus.* This tool showed high concordance (96%) with LPA for detecting clinically significant NTM species and incidentally found mixed species in NTM samples [23]. Here, we showed that this tool has moderate performance for identifying multiple NTM species in samples. However, this tool had the worst performance among those we assessed. For example, it misidentified mixed NTM due to *M. intracellulare* and *M. scrofulaceum* (that both belong to the *M. avium* complex) and *M. fortuitum* and *M. peregrinum* (that both belong to the *M. fortuitum* complex) as due to one species in each case. It also gave misidentifications when the proportion of the minor species was as low as 10%. In addition, this tool does not have the ability to detect the proportion of each species represented in the mixture. Thus, Mykrobe-Predictor, while reliable for diagnosis of mycobacteria [23], is not recommended for identification of mixed NTM species.

To extend the analysis to a real-world situation, we used WGS data from 16 clinical samples identified by LPA as likely representing mixed infections of NTM species [43,44]. In our study, MetaPhlAn3 detected the highest number (6/16 samples) of mixed NTM infections. However, the species identifications were only partially concordant with the LPA results, or disagreed completely. In addition, in three samples, the proportion of reads from the minor species was assessed as being lower than 0.2% that might not be the actual mixed NTM or clinically insignificant. Notably, the majority (75%) of the suspected mixed-NTM samples detected by LPA were reported to contain only single species by at least three out of the four WGS-analysis tools. Previously, Mykrobe-Predictor concordantly reported a small proportion of mixed species samples (2/25, 8%) compared to LPA [23]. In the majority of our suspected mixed samples (13/16, 81.25%), Mykrobe-Predictor identified only one species that was concordant with the LPA.

We further analyzed the samples using a metagenomic assembly approach. The one known mixed sample obtained from the public database contained two sequence types of *rpoB*, indicating a mixture of NTM species. However, only one sequence type of each gene (16S rRNA and *rpoB* gene) was obtained from most of clinical samples. Only one of our clinical sample was identified as mixed NTM species based on the *rpoB* sequence types found. Our failure to detect the *hsp65* gene from metagenomic assemblies might be due to the low quality of, or errors in the draft assemblies [45]. In some metagenomic assemblies, we could not detect the 16S rRNA gene. Gene prediction tools typically focus on complete rRNAs, but the 16S rRNA gene is relatively short (>1200 bp) and is commonly fragmented in sequencing reads [46]. Therefore, we concluded that the *rpoB* gene provides the best performance for identification of NTM species using the metagenomic assemblies analysis approach.

Notably, the metagenomics analysis approach was typically in agreement with at least two WGS-analysis tools but only in partial agreement with LPA. Due to the different analysis algorithms, the WGS-analysis approach seems to have higher sensitivity than the metagenomic analysis approach. The results from the latter suggested that there might be only single species of NTM present in suspected mixed-species samples according to LPA. Most of the WGS-analysis tools as well as the metagenomic analysis approach correctly identified the species present in the one known mixed NTM-species sample, which had been confirmed by conventional laboratory methods. In addition, the results from simulated mixed-species datasets indicate that WGS-analysis tools are reliable for detection and identification of mixed NTM species. Taken together, it is likely that LPA has low specificity to detect mixed NTM species in clinical samples. The discordance between LPA and WGS analysis might be due to nature of the genetic targets and their resolution. However, lack of a gold standard hampered our ability to reach a clear conclusion.

Limitations of our study should be noted. A relatively small number (*n* = 16) of clinical samples suspected to be cases of mixed NTM infection was used. Use of Sanger sequencing of the specific target gene(s) to confirm the identities of species in multiple colonies grown from a single clinical sample might be a gold standard to confirm the presence of mixed NTM infections when these are suggested by LPA. However, validation of the LPA results was not included in our study and this is considered a major limitation. Although we used LPA as the routine laboratory method for comparison, it can generate false-positive and false-negative results due to unsuccessful hybridization caused by heterogeneity within the probe-binding site [44]. It also has varying sensitivity for identification of multiple species [47]. Therefore, we used positive and negative controls derived from WGS datasets of known NTM species. A further limitation of LPA is the cross reactivity of some probes for the *M. avium*-*intracellulare-scrofulaceum* group, *M. fortuitum* complex and *M. intracellulare* Type 2, thus reducing specificity [48]. Although we have illustrated that WGS-analysis tools achieved excellent performance for diagnosis of mixed NTM infections in simulated datasets, we could not clearly illustrate their performance in real clinical isolates. Only a degree of concordance with LPA was demonstrated. Additional investigations should use a higher number of clinical mixed infections with a greater range of NTM species. Identification of species should be confirmed using various methods such as biochemical tests. WGS analysis should also be carried out on samples from colonies spiked with known species and on sequences derived from DNA extracted directly from clinical sample material.

## 4. Materials and Methods

### 4.1. Study Population: Clinical Samples of Mixed NTM Species

Twenty-three clinical culture samples of mixed infection with different species of NTMs from the biobank of Srinagarind Hospital, Khon Kaen Province, Thailand, collected during 2012–2016, were used in this study. The NTM species in these samples were identified by line-probe assay using INNO-LiPA MYCOBACTERIA v2 (INNOGENETICS GmbH, Heiden Germany) according to the manufacturer’s protocol [49].

### 4.2. Sample Preparation and WGS of Clinical Samples

Genomic DNA of the 23 samples was extracted from multiple loops of colonies using the cetyl-trimethyl-ammonium bromide-sodium chloride (CTAB) method [50]. All clinical samples of mixed NTM species infection were sent for sequencing by NovogeneAIT, Hong Kong, using the HiSeq (Illumina) platform generating 150-bp paired-end reads. Unfortunately, sequencing failed for 7 of the 23 samples because there was insufficient material. The characteristics of each sample (*n* = 16) are shown in Table 5.

### 4.3. In-Silico Simulated Samples Containing Various Proportions of Reads from Different NTM Species

Positive control datasets of mixed species of NTM were simulated from WGS data of known single NTM species. WGS data (FASTA format) from 14 known NTM species and one *M. tuberculosis* strain (HN-506) were retrieved from NCBI Genbank and the European Nucleotide Archive (ENA). These were used to provide the simulated mixed samples (2 × 150 bp based on Illumina HiSeq) using the ART simulator [51] with various mean depths of coverage (10×, 30×, 50×, 70×, 90× and 100×) across the genome. Then, 100 simulated datasets of mixed NTM species were produced by mixing of sequence reads from two different NTM species, with various percentages of reads from the first and second species (10/90, 30/70, 50/50, 70/30, and 90/10). Simulated reads of individual species (*n* = 15) with a mean coverage of 100× from the ART simulator were used as non-mixed (single species) samples of mycobacteria. WGS data in FASTA format and simulated mixed species of NTM samples are listed in Table S6.

### 4.4. WGS Data Samples from a Public Database

The WGS data of 14 species of NTM plus *M. tuberculosis* (in total 155 datasets), sequenced using the Illumina platform, were randomly selected from the Sequence Read Archive (SRA) database. One mixed sample from a bird, which was reported as a mixed infection with different species of NTM [24], was also included. The reads of each dataset were extracted using Fastq-dump from SRA Toolkit version 2.9.1 (http://ncbi.github.io/sra-tools/ accessed on 15 April 2021) [52]. The list of sequence samples is shown in Table S4.

### 4.5. Bioinformatics Analysis

#### 4.5.1. QC Check and Data Preparation of Sequence Reads

The quality of sequence reads obtained from clinical samples and the public database were checked by FastQC version 0.11.5 [53]. All sequence reads greater than 75 bp were retained. Reads shorter than 75 bp and potentially contaminating adapter sequences were removed by Trimmomatic version 0.36 [54] using the options LEADING:3 TRAILING:3 SLIDINGWINDOW:4:15 MINLEN:75. The filtered reads were then used in the downstream applications.

#### 4.5.2. Detection of Mixed Species of NTM Using Analysis of Read Frequencies Supporting SNP Alleles

This approach used the read frequencies supporting SNPs to distinguish the mixture of species in samples. Because of the common prevalence of mixed NTM infections [4] due to *M. intracellulare* and *M. abscessus*, simulated datasets based on different percentages of reads from these two species (10/90, 30/70, 50/50, 70/30, and 90/10) were selected for analysis using this method. All these datasets were mapped to both *M. abscessus* UC22 (GenBank Accession number: CP012044) and *M. intracellulare* FLAC0181 reference genomes (GenBank Accession number: CP023149.1) using BWA-MEM version 0.7.17 [55]. SAMtools version 0.1.19 [56] was used to convert and sort mapped sequences to the SAM-BAM format. Re-alignment of the mapped reads was done using GATK version 3.4.0 [57]. Variant calling and filtering were then performed to generate the intersection variant set between SAMtools and GATK. SAMtools pileup was used to generate the combined nucleotide frequency for each positional SNP. Outputs of this step were extracted to construct the graph of SNP allele frequencies using GraphPad Prism 8 (GraphPad, San Diego, CA, USA). Mixed infection was visually determined based on the pattern of heterozygous SNP allele frequencies in the samples across genome depending on the number and proportion of species/strains present.

#### 4.5.3. Detection of Mixed Species of NTM Using PubMLST

Before analysis with PubMLST [58], an assembled sequence file is required. De-novo assembly of the filtered reads for all samples was performed using SPAdes version 3.11.1 [59] (http://cab.spbu.ru/software/spades accessed on 15 April 2021). The quality of scaffold files was checked using QUAST version 5.0.2 [60] and then the files were used as the input for submission to the PubMLST web interface (https://pubmlst.org/ accessed on 15 April 2021). For samples apparently containing members of the *M. abscesuss* complex, subspecies present were identified using MAB-MLST (https://pubmlst.org/mabscessus/ accessed on 15 April 2021), which is an additional tool offered by the PubMLST webpage.

#### 4.5.4. Detection of Mixed Species of NTM Using MetaPhlAn3

MetaPhlAn3 [36] (https://github.com/biobakery/MetaPhlAn/wiki/MetaPhlAn-3.0 accessed on 15 April 2021) was used. The paired-end filtered reads of samples were used as the input for the MetaPhlAn3 classification analysis with default parameters. The mpa_v30_CHOCOPhlAn_201901 database was used for the analysis.

#### 4.5.5. Detection of Mixed Species of NTM Using Kraken2

Detection of mixed NTM species from samples was done using Kraken2 [28] with the Maxikraken2 database and *k-mers* approach. NTM species identification of each paired-end read was done using the options –use-names and –report, which provided the taxonomic names associated with each classified sequence and standard ranks for each taxon. Next, Bracken tool [61] was used to estimate genus- and species-level abundance using output from Kraken2 classification results as the input.

#### 4.5.6. Detection of Mixed Species of NTM Using Mykrobe-Predictor

Mykrobe-Predictor [42] (https://github.com/Mykrobe-tools/mykrobe accessed on 15 April 2021) was used for mycobacteria species identification. The sequence reads were used to identify mycobacteria species by default parameters.

#### 4.5.7. Detection of Mixed Species of NTM Using Metagenomic Assembly Analysis Based on 16S rRNA, *rpoB* and *hsp65* Genes

The metagenomic assemblies of the samples were called using Prokka 1.13.7 [62]. The sequences of 16S rRNA, *rpoB* and *hsp65* genes used for NTM species identification [14] were extracted and compared to those in the GenBank database using the BLAST algorithm (http://blast.ncbi.nlm.nih.gov/Blast.cgi accessed on 15 April 2021). Multiple-sequence alignment (MSA) was performed using Seaview version 5 [63]. The presence of distinctly different sequence types from a single sample in the MSA was taken as evidence of mixed NTM species.

### 4.6. Statistical Analysis

Since there is no gold-standard method for detecting and identifying species in mixed infections of NTM species, simulated sequence reads from each of the two NTM species retrieved from reference and publicly available genome sequences were used as positive controls to calculate sensitivity. Sequence reads based on genomes of single NTM species and sequence reads from public databases were used as negative control to calculate specificity. The sensitivity and specificity of the various WGS-analysis tools for identification of mixed infection with different NTM species were calculated. Sensitivity = true positive/(true positive + false negative). Specificity = true negative/(true negative + false positive).

## 5. Conclusions

We evaluated diagnostic performance of WGS-analysis tools for identification of mixed NTM species infection. Kraken2 provided the highest sensitivity and specificity for this, together with accurate estimation of relative abundance of each species in the samples. PubMLST and MetaPhlAn3 had slightly lower performance but might be useful ancillary methods. LPA seems to have inadequate performance to detect mixed NTM species infection in our study. Accurate species identification will assist choice of appropriate treatment and reduce the mortality rates caused by NTM infection.

## Figures and Tables

**Table 1:** Ability of different WGS-analysis approaches to detect NTM species represented in simulated datasets containing reads from two different NTM species in various ratios (*n* = 100) *^a^*.

Species Identification Types	PubMLST (*n* = 100)	MetaPhlAn3 (*n* = 100)	Kraken2 (*n* = 100)	Mykrobe-Predictor (*n* = 100)
Correct mixed species	99 (99%)	95 (95%)	100 (100%)	58 (58%)
Incorrect mixed species	0	5 (5%) *^b^*	0	25 (25%)
Single species only	1 (1%)	0	0	17 (17%)

*^a^* Each combination of species at different proportions was considered as an individual sample in the total of 100 samples. *^b^* This tool detected *M. tuberculosis* and *M. canettii*, both belonging to the *M. tuberculosis* complex.

**Table 2:** Comparison of WGS-analysis tools for species-level identification of NTMs in a single mixed-species dataset from a previous study (RA%: relative abundance of reads in the dataset).

Accession Number	WGS-Analysis Tools							Metagenomic Assembly Analysis
	PubMLST		MetaPhlAn3		Kraken2		Mykrobe-Predictor	BLAST (*rpoB* Gene)
	Species	RA (%)	Species	RA (%)	Species	RA (%)	Species	Species
SRR5043021 [25]	*M. hassiacum,* *M. peregrinum*	49 39	*M. hassiacum,* *M. peregrinum*	83.67 16.33	*M. hassiacum,* *M. peregrinum*	73.41 24.29	*M. hassiacum* *	*M. hassiacum,* *M. peregrinum*

* Mykrobe-Predictor has no feature for proportion identification.

**Table 3:** Ability of WGS-analysis approaches to identify NTM species present (single-species control) in datasets from a public database.

Species Identification Types	PubMLST (*n* = 155)	MetaPhlAn3 (*n* = 155)	Kraken2 (*n* = 155)	Mykrobe-Predictor (*n* = 155)
Correct single species	149 (96.12%)	128 (82.58%)	152 (98.06%)	149(96.12%)
Mixed species	4 (2.58%)	26 (16.77%)	3 (1.93%)	0
Incorrect species	1 (0.64%)	0	0	5 * (3.22%)
No result	1 (0.64%)	1 (0.64%)	0	1(0.64%)

* One sample was only identified to the genus level (*Mycobacterium* sp.).

**Table 4:** Performance of WGS-analysis tools for identifying multiple species in datasets of reads from NTMs.

Sample Groups	Number of Samples (Correct Species Identification/Total Samples)			
	PubMLST	MetaPhlAn3	Kraken2	Mykrobe-Predictor
Simulated mixed species *^a^*	99/100	95/100	100/100	58/100
Mixed species from public database	1/1	1/1	1/1	0/1
**Sensitivity of tool**	**99%** (**100/101**)	**95.04%** (**96/101**)	**100%** (**101/101**)	**57.42%** (**58/101**)
Simulated single species control	15/15	14/15	15/15	14/15
Single species control from public database	149/155	128/155	152/155	149/155
**Specificity of tool**	**96.47%** (**164/170**)	**83.52%** (**142/170**)	**98.23%** (**167/170**)	**95.88%** (**163/170**)

*^a^* Each combination of species at different proportions was considered as individual sample in the total of 100 samples.

**Table 5:** List of clinical samples of mixed infection with different species of mycobacteria detected by line-probe assay (LPA).

Sample ID	NTM Species (According to LPA)	Date of Collection	Sex	Age	Specimen Types
MIX80105	*M. abscessus* subsp. *massiliense, M. abscessus*	10 January 2014	M	43	Sputum
MIX80487	*M. intracellulare, M. scrofulaceum*	16 February 2015	M	49	Nasal Cavity (swab)
MIX80628	*M. gordonae, M. simiae*	19 February 2014	M	34	Bone marrow
MIX80885	*M. avium, M. intracellulare*	12 March 2014	M	63	Sputum
MIX81256	*M. kansasii, M. intracellulare*	8 April 2014	F	50	Bronchial wash
MIX81523	*M. kansasii, M. malmeonse*	8 August 2016	F	56	Sputum
MIX81666	*M. tuberculosis* complex, *M. scrofulaceum*	8 July 2016	M	69	Sputum
MIX82390	*M. intracellulare, M. avium*	15 July 2014	M	34	Bone marrow
S12260	*M. fortuitum, M. peregrinum*	22 August 2016	F	75	Blood
S80510	*M. gordonae, M. fortuitum*	17 February 2015	M	57	Sputum
S81158	*M. kansasii, M. scrofulaceum*	29 April 2013	M	28	Sputum
S81463	*M. simiae, M. fortuitum*	29 May 2013	F	61	Sputum
S81801	*M. fortuitum, M. peregrinum*	27 July 2016	F	47	Pus
S82945	*M. intracellulare, M. abscessus*	2 October 2013	F	81	Sputum
S83359	*M. intracellualre, M. fortuitum*	10 October 2014	F	33	Sputum
S83411	*M. kansasii, M. avium*	5 November 2013	M	75	Sputum

**Table 6:** Comparison between WGS analysis and LPA for detection and identification of mixed NTM species infection in Thai clinical samples.

Sample ID	LPA	WGS-Analysis Tools			
		PubMLST (MAB-MLST) (%RA)	MetaPhlAn3 (%RA)	Kraken2 (%RA)	Mykrobe-Predictor
MIX80105	*M. abscessus* subsp. *massiliense,* *M. abscessus*	*M. abscessus* (*M. abscessus* subsp. *massiliense*)	*M. abscessus*	*M. abscessus*	*M. abscessus*
MIX80487	*M. intracellulare, M. scrofulaceum*	*M. malmoense*	*M. malmoense*	*M. malmoense*	*M. parascrofulaceum*
MIX80628	*M. gordonae, M. simiae*	*M. sherrisii,* *M. asiaticum * (96, 3)	*M. sherrisii, M. asiaticum* *M. simiae * (99.83, 0.16, 0.01)	*M. sherrisii*	*M. sherrisii*
MIX80885	*M. avium, M. intracellulare*	*M. asiaticum*	*M. * sp. *1165178.9, M. lepraemurium*, *M. colombiense * (99.91, 0.06, 0.03)	*M.* sp. *1165178.9*	*M. intracellulare*
MIX81256	*M. kansasii, M. intracellulare*	*M. intracellulare*	*M. intracellulare*	*M. intracellulare*	*M. intracellulare*
MIX81523	*M. kansasii, M. malmeonse*	*M. attenuatum*	*M. kansasii*	*M.* sp. *MK136 ^a^*	*M. kansasii*
MIX81666	*M. tuberculosis* complex, *M. scrofulaceum*	*M. tuberculosis*	*M. canettii ^b^, M. * sp. *E3198* *M. * sp *852002-50816 SCH5313054-b* (96.70, 1.85, 1.45)	*M. tuberculosis*	*M. tuberculosis* *M. intracellulare*
MIX82390	*M. intracellulare, M. avium*	*M. scrofulaceum*	*M. lepraemurium, M. scrofulaceum * (75.94, 24.06)	*M. * sp. *ACS4054,* *M. scrofulaceum* (9.23, 7.13)	*M. intracellulare*
S12260	*M. fortuitum, M. peregrinum*	*M. fortuitum*	*M. fortuitum*	*M. fortuitum*	*M. fortuitum*
S80510	*M. gordonae, M. fortuitum*	*M. abscessus* (UD)	*M. gordonae, M. abscessus* (81.26, 18.74)	*M. gordonae*	*M. gordonae*
S81158	*M. kansasii, M. scrofulaceum*	*M. attenuatum*	*M. kansasii*	*M.* sp. *MK136 ^a^*	*M. kansasii*
S81463	*M. simiae, M. fortuitum*	No result	*M. rhodesiae*	*M.* sp. *M26 ^c^*	*M. farcinogenes*
S81801	*M. fortuitum, M. peregrinum*	*M. fortuitum*	*M. fortuitum, M. abscessus* (99.99, 0.01)	*M. fortuitum*	*M. fortuitum*
S82945	*M. intracellulare, M. abscessus*	*M. abscessus* (*M. abscessus* subsp. *massiliense*)	*M. abscessus*	*M. abscessus*	*M. abscessus*
S83359	*M. intracellulare, M. fortuitum*	*M. fortuitum*	*M. fortuitum*	*M. fortuitum*	*M. fortuitum*
S83411	*M. kansasii, M. avium*	*M. persicum*	*M. persicum*	*M. persicum*	*M. kansasii*
**Single species** *^d^*	**0**	**14** (**87.5%**)	**10** (**62.5%**)	**15** (**93.75%**)	**15** (**93.75%**)
**Mixed species** *^e^*	**16**(**100%**)	**1** (**6.25%**)	**6** (**37.5%**)	**1** (**6.25%**)	**1** (**6.25%**)
**No result**	**0**	**1** (**6.25%**)	**0**	**0**	**0**
**Total**	**16**	**16**	**16**	**16**	**16**

**Note:** MAB-MLST refers to *M. abscessus* complex-multilocus sequence typing, UD refers to un-differentiable, *^a^* *M.* sp. *MK136* reported by the tool refers to *M. attenuatum, ^b^ M. canettii* is a member of the *M. tuberculosis* complex, *^c^ M.* sp. *M26* reported from the tool refers to *M. massilipolynesiensis.* %RA refers to relative abundance of different species within mixed samples. Red text highlights samples in which the WGS-analysis tool identified multiple species of NTM, and the relative proportions of each estimated by the tool. *^d^* Number of samples in which the tool claimed only a single species was present. *^e^* Number of samples in which the tool identified two species of NTM.

## Data Availability

The WGS data for 16 samples are available in the Sequence Read Archive (https://www.ncbi.nlm.nih.gov/sra accessed on 15 April 2021) with the Accession No. PRJNA716753.

## Supplementary Materials

The following are available online at https://www.mdpi.com/article/10.3390/pathogens10070879/s1, Figure S1: Graph of heterozygous SNP frequencies for five different proportions of simulated mixed NTM species samples (*M. intracellulare* mixed with *M. abscessus*) mapped against each reference genome *M. intracellulare* and *M. abscessus*, respectively; Table S1: simulated datasets of sequence reads representing two NTM species in various proportions and the identifications made by four WGS-analysis tools; Table S2: WGS analysis tools for in silico simulated non-mixed (single species) NTM species identification; Table S3: two different sequence types of *rpoB* gene were identified by metagenomic assembly analysis of a known mixed NTM dataset (SRR5043021) and similar analysis of a Thai clinical sample; Table S4: summary results of WGS analysis for identifying NTM species based on data from a public database; Table S5: comparison between WGS-analysis tools, metagenomic assembly analysis (16S rRNA and *rpoB* gene) and LPA for detection and identification of mixed NTM species infection in known mixed-NTM samples from a public database and Thai clinical samples; Table S6: sources of WGS data and simulated mixed species of NTM samples.
