# Supplementary figures and images for: Whole-Genome Sequencing Analysis to Identify Infection with Multiple Species of Nontuberculous Mycobacteria
Source: Pathogens. 2021 Jul 11;10(7):879. doi: 10.3390/pathogens10070879 (PMC8308675; doi:10.3390/pathogens10070879)

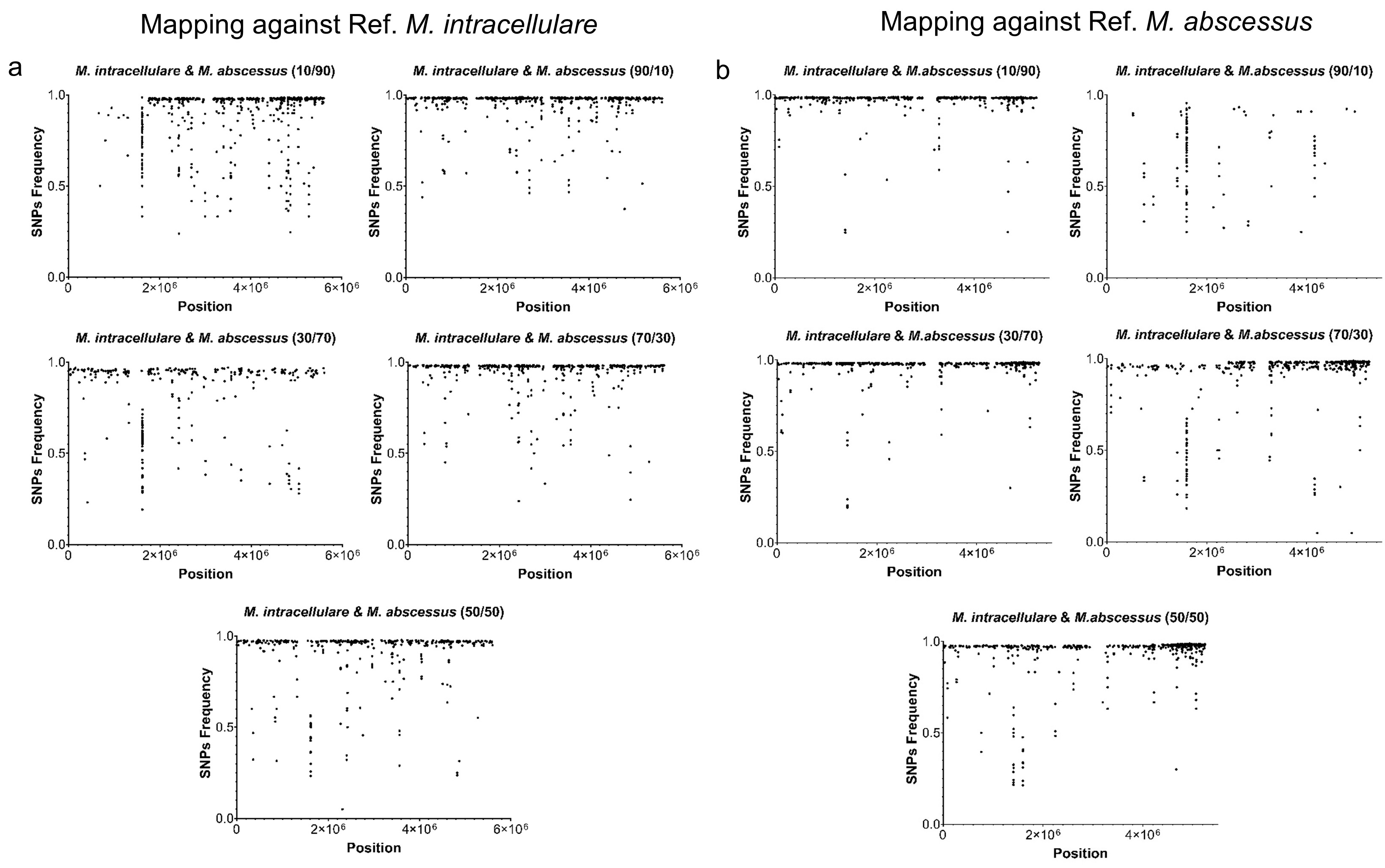

Supplement: Supplementary file 1 [file pathogens-10-00879-s001.zip › pathogens-1217905 - supplementary figure.jpg]
